# Lipid‐based gene delivery to macrophage mitochondria for atherosclerosis therapy

**DOI:** 10.1002/prp2.584

**Published:** 2020-04-01

**Authors:** Felix H. Zakirov, Dongwei Zhang, Andrey V. Grechko, Wei‐Kai Wu, Anastasia V. Poznyak, Alexander N. Orekhov

**Affiliations:** ^1^ I. M. Sechenov First Moscow State Medical University (Sechenov University) Moscow Russian Federation; ^2^ Diabetes Research Center Traditional Chinese Medicine School Beijing University of Chinese Medicine Beijing China; ^3^ Federal Research and Clinical Center of Intensive Care Medicine and Rehabilitology Moscow Russian Federation; ^4^ Department of Internal Medicine National Taiwan University Hospital Taipei Taiwan; ^5^ Institute for Atherosclerosis Research Skolkovo Innovative Center Moscow Russia; ^6^ Institute of Human Morphology Moscow Russia; ^7^ Laboratory of Angiopathology Institute of General Pathology and Pathophysiology Moscow Russia

**Keywords:** atherosclerosis, gene delivery, liposomes, macrophages, mitochondrial dysfunction, mtDNA damage

## Abstract

Atherosclerosis with associated cardiovascular diseases remains one of the main causes of disability and death worldwide, requiring development of new solutions for prevention and treatment. Macrophages are the key effectors of a series of events involved in atherogenesis, such as inflammation, plaque formation, and changes in lipid metabolism. Some of these events were shown to be associated with mitochondrial dysfunction and excessive mitochondrial DNA (mtDNA) damage. Moreover, macrophages represent a promising target for novel therapeutic approaches that are based on the expression of various receptors and nanoparticle uptake. Lipid‐based gene delivery to mitochondria is considered to be an interesting strategy for mtDNA damage correction. To date, several nanocarriers and their modifications have been developed that demonstrate high transfection efficiency and low cytotoxicity. This review discusses the possibilities of lipid‐based gene delivery to macrophage mitochondria for atherosclerosis therapy.

**Abbreviations**
ASCVD: atherosclerotic cardiovascular disease
CRP: C‐reactive protein
NA: nucleic acid
NP: nanoparticle
SAMS: statin‐associated muscle symptoms

## 1 INTRODUCTION

Atherosclerosis is a multifactorial disease, which is characterized by the formation of fatty plaques in the arterial wall and chronic inflammatory response. Atherosclerosis leads to narrowing of the lumen of the affected vessel and increases the risk of thrombosis, which can be followed by lethal events, such as ischemic stroke and sudden cardiac death.^1^ ^,^ ^2^ It is well‐known that atherosclerosis development can be a result of acquired and inherited factors. To date, numerous genetic variations and mutations have been shown to predispose humans to atherogenesis. Among the risk factors of atherosclerosis are dyslipidemia, arterial hypertension, diabetes mellitus, and old age.^3^ Monocytes and macrophages play leading roles at all stages of atherosclerosis development, contributing to the local inflammatory response, cholesterol accumulation, and plaque growth.^4^

Modern advances of genetics and molecular biology have improved our understanding of atherosclerosis pathogenesis and opened new perspectives for developing diagnostic and therapeutic approaches.^5^ It is evident that treatment of atherosclerosis requires implementation of complex approaches that employ a combination of physical, chemical, and biological methods, such as those provided by nanomedicine.^6^

Mitochondrial dysfunction caused by mitochondrial DNA (mtDNA) damage is a well‐studied cause of cell dysfunction and death observed in atherosclerosis.^7^ Correspondingly, dysfunctional mtDNA is regarded as a promising target for atherosclerosis treatment strategies. The latest tendencies of treatment of mitochondrial diseases include direct nucleic acid (NA) delivery into these organelles. Currently, specially developed nanocarriers for DNA delivery into the mitochondria demonstrate sufficient efficacy and low cytotoxicity.^9^ Lipid‐based nanocarriers are currently well investigated and widely used in research and therapy.^10^ Being constantly improved and upgraded, liposomes often demonstrate biocompatibility and transfection efficiency, which makes them an advantageous agent for immunostimulation, drug, and NA delivery not only to cells but also to mitochondria.^11^ The aim of this review is to summarize current knowledge on the use of lipid‐based nanocarriers for targeted gene delivery to macrophage mitochondria and assess its role in future atherosclerosis therapy.

## 2 CURRENT CARDIOVASCULAR DISEASE MANAGEMENT STRATEGIES

Current approaches for treatment of atherosclerosis are mainly focused on lipid lowering with statins, reduction in risk of thrombosis with anticoagulants, and alleviation of inflammation by means of immunomodulation.^12^, ^13^ During the recent years, substantial progress was made in the improvement of atherosclerosis treatment. Currently, a number of therapeutic agents applicable for atherosclerotic cardiovascular disease (ASCVD) treatment are being used. Having proved efficacy in atherosclerosis therapy, some of these agents can be associated with high residual cardiovascular risk, low cost‐efficiency, various contraindications, and side effects. Among them, lipid lowering statin drugs, fibrates, PCSK‐9 inhibitors, and niacin can be highlighted as important therapeutic agents that deserve to be discussed in detail.

Statins (HMG‐CoA reductase inhibitors) are used for the first‐line treatment for ASCVD reduction. These drugs can be used in different groups of patients providing low‐density lipoprotein‐cholesterol (LDL‐C) lowering effect of desirable intensity (low, medium, or high) with up to more than 50% LDL‐C reduction.^14^ While lipid lowering effect can be regarded as the primary effect of statins, they also have secondary effects (so‐called pleiotropic effects) that can contribute to ASCVD management. Importantly, anti‐inflammatory activity of statins is well‐known. It was shown that statins reduce the levels of proinflammatory cytokines and C‐reactive protein (CRP), which is a nonspecific, but highly sensitive biomarker of inflammation.^15^, ^16^ Moreover, clinical studies reported statins to stabilize the plaque and reduce vascular wall inflammation.^17^

However, statins are not free from adverse effects, drug interactions, and intolerance in some patients.^18^ That is why clinician‐patient risk discussion as well as risk‐benefit assessment are recommended before statin prescription.^19^ Despite this fact, in some cases it is still necessary to stop statin therapy, mainly because of statin‐associated muscle symptoms (SAMS), which can vary from myalgia to rhabdomyolysis and associated disorders.^20^ Therapeutic alternatives to statins have been considered, and several other drug classes have been developed, some of them being able to potentiate the ongoing statin therapy.

Fibrates (agonists of peroxisome proliferator‐activated receptor‐α (PPAR‐α)) are another group of medications that belong to nonstatin therapy, mainly aimed on triglyceride level control.^21^ It has also been reported that fibrates can be used in combination with statins to correct dyslipidemia and residual cardiovascular risk.^22^ The results of some studies have indicated anti‐inflammatory potential of fibrates.^23^ Nevertheless, the results of studies assessing the effect of fibrates on human lipid profile are sometimes contradictory.^24^ Moreover, presence of adverse effects is also notable, including nausea, myositis, and gallstones.^25^ Obviously, more studies are needed to assess the efficacy of fibrates and associated drug combinations in different groups of patients.

Recently, a new group of drugs, PCSK9 (proprotein convertase subtilisin/kexin type 9) inhibitors, came to clinical practice. The mechanism of action of these drugs relates to the reduction in the plasma level of PCSK9, therefore reducing its binding to low‐density lipoprotein receptor (LDLR). Using of PCSK9 inhibitors opens new opportunities for high‐risk patients, and is recommended in cases when general LDL‐lowering therapy is not sufficient.^26^ However, these drugs have their limitations. Firstly, the relatively high price of treatment with PCSK9 inhibitors makes them cost ineffective and hinders their widespread use.^27^ Secondly, little is known to date about the long‐term tolerance of these drugs due to their recent approval.^28^ Future studies will add more detail on the PCSK9 inhibitors safety and effectiveness, allowing for a better positioning of these drugs within the spectrum of antiatherosclerosis and antihyperlipidemia treatments.

Niacin was one of the first effective lipid‐lowering agents that have been discovered, which acts through LDL and HDL lowering in the plasma.^29^ However, clinical trials did not confirm a beneficial effect on niacin on cardiovascular events incidence, while the drug's side effects, including skin, gastrointestinal, and musculoskeletal effects, have been observed. Thus, there are currently no medications with niacin approved in Europe, and no recommendations to use it in the US guidelines.^30^, ^31^

Modern guidelines for cardiovascular disease prevention and therapy suggest low‐lipid diet and active lifestyle as primary measures to reduce cardiovascular risk. Dietary supplements also have their place as risk‐reducing agents helping to normalize the blood lipid profile. Omega‐3 fatty acids, according to the results of several trials, were shown to reduce plasma triglycerides, and were associated with the reduction on fatal and nonfatal cardiovascular events.^32^, ^33^ The most stable eicosapentaenoic acid, icosapent ethyl, is currently indicated to reduce overall cardiovascular risk and is used as adjuvant to statin and nonstatin therapy.^34^

Accumulating clinical evidence suggests that existing lipid‐lowering strategies alone may not be sufficient to eliminate the cardiovascular risk. These results encouraged multiple studies to investigate the best treatment combinations (statin + nonstatin approaches) in order to lower the cardiovascular risk in a more reliable way.^35^, ^36^ Generally, various dyslipidemias that can occur in different groups of patients are mentioned as the main cause of residual cardiovascular events.^37^ At the same time, chronic inflammation, which plays a key role in atherosclerosis development, is likely to be a prominent risk factor of cardiovascular events. Despite the improvements achieved in clinical practice recently, atherosclerosis still remains a serious cause of disability and death.^38^ Future studies are needed to identify the optimal therapeutic strategies that allow for a more profound and stable reduction in cardiovascular risk in different groups of patients. Moreover, novel solutions are needed to combat the development of atherosclerosis by effectively preventing the formation of new plaques and induce regression of the existing plaques.

## 3 NANOTECHNOLOGIES FOR ATHEROSCLEROSIS THERAPY

One of the most recent treatment strategies of atherosclerosis is nanomedicine, which comprises chemical, biological, and physical technological applications.^39^ Several novel techniques, including nanoparticle (NP) target therapy, drug delivery, nanovisualization, have been introduced and are being tested. As atherosclerosis is a multifactorial disease, there are number of possible targets for nanomedical methods in the atherosclerotic plaque: extracellular matrix, endothelial cells, and macrophages.^40^ Macrophages are considered to be a promising target mainly due to the possibility of effective NP targeting.^10^ NP design can provide combinative approaches for targeted treatment of atherosclerosis by carrying therapeutic agents, such as lipid‐lowering and anticoagulant drugs, siRNA, and DNA plasmids. Delivery of such agents directly into the target cells showed antiatherogenic effects in vitro as well as in vivo.^41^, ^42^ In addition, NPs can be used for accurate visualization of vessels and plaques.^43^

Novel drug delivery strategies include methods of selective targeting of mitochondria with lipid‐based carriers. This approach is aimed at reducing mitochondrial dysfunction and alleviating mtDNA damage, which contributes to the development of pathological conditions in atherosclerosis.^44^

## 4 ROLE OF MACROPHAGES IN ATHEROSCLEROSIS

Several recent studies conducted in vitro on animal models (mostly ApoE^−/−^ and LDLR^−/−^ mice) and human rupture plaques have determined the critical role of macrophages in atherosclerosis pathogenesis.^45^ In growing atherosclerotic plaques, macrophages actively participate in lipid accumulation giving rise to foam cells and expanding the plaque. Moreover, macrophages also contribute to the immune response by releasing cytokines and chemokines providing the inflammatory component of atherosclerosis.^46^ In atherosclerosis, the heterogeneity of monocytes/macrophages is shifted toward the prevalence of proinflammatory activation.^47^ Proinflammatory or M1 macrophages release tumor necrosis factor‐alpha (TNF‐α), interleukins, and chemokines, and also produce high levels of ROS and nitric oxide (NO).^48^ At the same time, anti‐inflammatory M2 macrophage phenotype is characterized by IL‐10 and IL‐1 receptor agonist secretion and may induce plaque regression and tissue repair.^49^

Macrophages are involved in lipid metabolism via cholesterol efflux and oxidized LDL (oxLDL) uptake.^50^ OxLDL particles are internalized through interaction with macrophage scavenger receptors (SR) (CD36, SR‐A1, and lectin‐like oxLDL receptor‐1 (LOX)) and then processed in the lysosomes. Subsequently, free cholesterol is released from the macrophages via ABC (ATP‐binding cassette) transporters (ABCA1 and ABCG1) facilitating the formation of HDL and removing the excess cholesterol. However, when the cholesterol efflux system is dysregulated due to excess plasma lipid levels, foam cells develop, which is believed to be a hallmark of atherosclerosis.^51^ In the arterial wall, macrophages participate in the immune response via a number of cell receptors, such as Toll‐like receptors (TLR), mannose‐receptor (MR), SRs, and Fc receptors.^52^ Some of these molecules are currently considered as potential targets for atherosclerosis therapy.

## 5 TARGETING OF MACROPHAGES RECEPTORS

A number of known macrophage surface receptors have been considered as potential targets for molecular therapeutic approaches.^10^, ^52^, ^53^ NPs containing ligands for these receptors demonstrate significantly higher selectivity and transfection efficiency.^54^, ^55^, ^56^ The mannose receptor (MR), which is a C‐type lectin I transmembrane protein, is believed to be a promising target for nanocarriers.^57^ The MR is expressed on dendritic cells, tissue macrophages, and liver sinusoidal endothelial cell.^58^ The MR plays an important role in antigen presentation, inflammatory response, and endocytosis, and can recognize the residues of mannose, fucose, and N‐acetylglucosamine. Incorporation of this ligands into the liposomes was shown to demonstrate high transfection efficiency and low cytotoxicity.^59^ In atherosclerotic plaque, high expression of MR is present in macrophages of the fibrous cap, while in macrophages of the lipid core, where the most of mtDNA alterations occur, expression is lower.^60^ Together, with presence of MR in other cell types, this target needs further investigation.

Another class of surface proteins that can be used for targeted drug delivery is integrins that play a crucial role in cell adhesion. Integrins can recognize proteins, components of extracellular matrix, phospholipids, as well as various amino acid sequences.^61^ Integrins are heterodimeric proteins, with each subunit containing ligand‐binding sites. Conjugation of certain peptides to nanocarriers demonstrated increased cellular uptake through integrin‐mediated pathway.^62^, ^63^

Pattern recognition receptors (PRRs) can be activated by damaged cell fragments or debris (damage‐associated molecular patterns, or DAMP) and bacterial LPS or DNA (pathogen‐ associated molecular patterns, or PAMP) (Schiltze and Schmidt, 2015). Toll‐like receptors (TLR) are one of the most studied family of receptors, which may be used for drug targeting and immunostimulating therapy.^64^, ^65^ However, the activation of PRRs is associated with inflammatory response and leads to T‐cell activation and cytokine release, which is obviously an undesirable event in atherosclerosis.^66^

## 6 MITOCHONDRIAL DNA DAMAGE

Mitochondria play a crucial role in regulating cell death, generating ROS, and maintaining cell metabolism and growth.^67^ The mitochondrial genome contains 2‐10 copies of mtDNA, each of them consisting of 16 569 base pairs and encoding 37 genes, including ETC (electron transport chain) components, tRNAs and rRNAs.^68^ Unlike nuclear DNA, mtDNA is not protected by histones and lacks efficient repair mechanisms, being therefore more susceptible for damage and accumulation of mutations, which increases with age. It is worth mentioning that mitochondrial dysfunction usually occurs only if more than 80% is damaged.^69^ One of the factors promoting mtDNA damage is constant ROS generation during the oxidative phosphorylation in the inner mitochondrial membrane.

Mitochondrial genome defects are well investigated and often associated with so‐called mitochondrial diseases.^70^, ^71^ However, mtDNA damage occurs in other diseases, including atherosclerosis, that can also be associated with mitochondrial dysfunction.^72^ Both animal and human studies showed that damaged mtDNA is more common in the sites of atherosclerotic lesions. Moreover, mitochondria appear to be considerably affected in macrophages, thus contributing to a number of atherogenesis events.^73^, ^74^

## 7 MITOCHONDRIAL DNA DAMAGE IN MACROPHAGES

Although mtDNA damage, in general, believed to be caused by excessive ROS generation in the mitochondria, recent in vivo studies demonstrated occurrence of mtDNA defects in atherosclerosis independently from oxidative stress. These studies were conducted in *apoE^−/−^* mice with downregulated polymerase‐γ (polG) proofreading activity. These mice showed high rate of mtDNA damage without increase in ROS and oxidative phosphorylation intensity. In comparison to classical *apoE^−/−^* models, polG‐deficient mice had increased hyperlipidemia and atherosclerosis. Moreover, *polG^−/−^/apoE^−/−^* monocytes were characterized by increased inflammatory cytokine secretion. These findings confirm possible development of atherosclerotic plaques and vessel damage promoted by damaged mtDNA with no associated ROS increase.^75^

A number of studies reported apoptosis of macrophages and vessel smooth muscle cells (VSMC) induced by mitochondrial dysfunction.^76^, ^77^, ^78^ As mentioned above, mitochondrial dysfunction can often be a result of accumulated mtDNA damage, subsequently leading to ROS generation and membrane defects. These conditions can stimulate the release of cytochrome C, an important cell death regulator, and promote apoptosis.^79^ Macrophage apoptosis in atherosclerotic plaques contributes to the necrotic core formation thus reducing the plaque stability and promoting thrombogenesis.^80^

The inflammatory response associated with atherosclerosis can be stimulated by endogenous antigens such as damaged mtDNA.^81^ According to the results of recent studies, a number of events can contribute to this process.^82^ The activation of TLRs under mitochondrial oxidative stress induces the NF‐κB pathway, which facilitates further immune response. It was also shown that the NF‐κB pathway in the atherosclerotic lesions’ macrophages promoted monocytes infiltration and plaque development.^83^ Moreover, oxidized mtDNA, which escaped degradation by autophagy, was reported to activate the NLRP3 inflammasome thus regulating the release of cytokines, such as IL‐1β and IL‐18.^84^, ^85^ In addition, mitochondrial dysfunction was also shown to affect the cholesterol efflux in macrophages.^86^ As this process is maintained by ATP‐dependent ABCA1 and ABCG1 transporters, the impaired ATP synthesis associated with mitochondrial dysfunction can inhibit the cholesterol efflux, therefore, disturbing lipid metabolism.^87^ Moreover, ABC transporters were also shown to mediate about 70% of the cholesterol efflux from the foam cells,therefore, their inhibition further facilitates foam cells formation.^88^

## 8 LIPID CARRIERS FOR GENE DELIVERY TO MITOCHONDRIA

One of the latest nanomedical tendencies of targeted therapy of mitochondrial dysfunction is using nanocarriers for gene delivery directly to the mitochondrion. This strategy aims to correct the mtDNA damage.^89^ Implementation of this strategy requires overcoming of several obstacles. First of them is the presence of two negatively charged mitochondrial membranes. While the outer membrane is quite similar to the cellular membrane by its composition, the inner membrane contains cardiolipin, which makes it impermeable for hydrophilic molecules. In order to pass this obstacle, the carrier must contain some hydrophobic and positively charged ligands.^90^, ^91^ Another challenge for targeted drug delivery to the mitochondria is endocytosis. To escape from the endosome, the carriers must be designed to contain ligands facilitating such transport.^92^

As mentioned above, accumulation of mtDNA damage contributes greatly to mitochondrial dysfunction as well as in atherogenesis. As mitochondrial genome consists of only 37 genes, it becomes possible to identify the potential targets for gene therapy in atherosclerosis. According to studies on ruptured plaques, arterial intima, and blood samples, a number of coding and noncoding mitochondrial genes, if mutated or damaged, were shown to cause various cell impairments and to be associated with atherogenesis. Among them are ETC proteins (NADH dehydrogenase, ATP synthase, cytochrome b, and cytochrome c oxidase subunits) and tRNA genes.^93^, ^94^, ^95^ Transfection of these genes may result in decrease in plaque progression and atherosclerotic lesion development.

**Figure 1:**
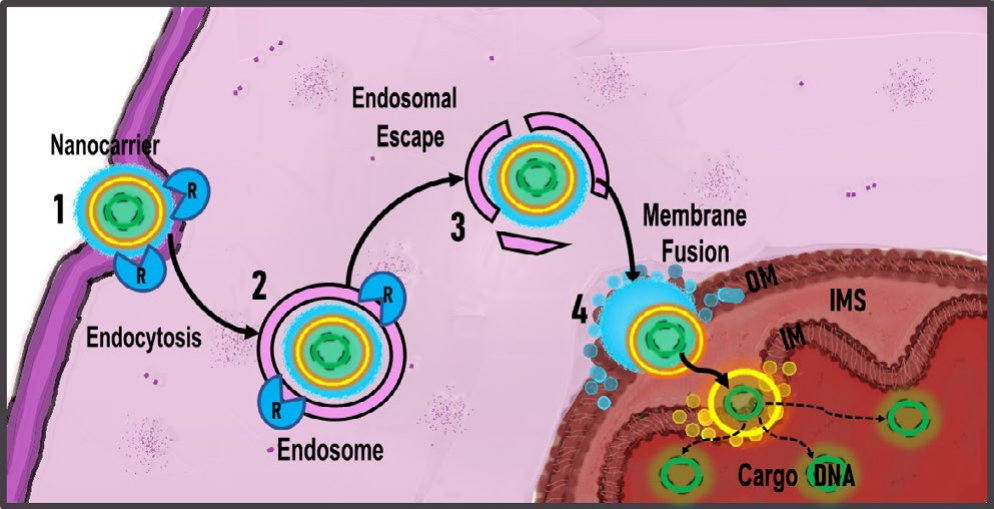
Lipid‐based mitochondria gene delivery mechanism**.** 1 – absorption of lipid carrier on macrophage surface; 2 – receptor‐mediated endocytosis of the carrier; 3 – endosome formation; 4 – endosomal escape and migration to mitochondrion; 5 – mitochondrial membrane fusion and cargo DNA release

Currently, a wide diversity of transport systems is known, including physical, chemical, biological, and combinatorial approaches. Several comparative analyses have been conducted to assess the toxicity, efficiency, and specificity of different methods of gene delivery into the mitochondria. Although all of them were far from implementation into the clinical practice, some of the methods demonstrate low cytotoxicity and high efficiency.^96^, ^97^, ^98^ The most promising technology is probably the use of lipid‐based nanocarriers. Such lipid carriers can be extensively modified to lower cytotoxicity and increase selectivity of delivered NA.^99^, ^100^ As well as in classical concept, any liposome contains lipid bilayer and aqueous core, which allow the carrier to fuse with cell membrane and subsequently release its content.^101^ However, this mechanism is obviously not enough for mitochondrial delivery. According to that, firstly endocytosis should be involved, followed by endosome formation and further endosomal escape. Only after being released from the endosome, the carrier will be able to go through both outer and inner mitochondrial membranes.^102^ Thus, successful gene delivery to mitochondria must include: 1 adsorption of liposomes on the cell surface,2 carrier endocytosis with endosome formation; 3 endosomal escape and migration to mitochondrion; and 4 mitochondrial membrane fusion and therapeutic agent release (Figure 1).^103^

Successful gene delivery to the mitochondria by lipid‐based nanocarriers depends on the proper design of the carriers, including the appropriate molar ratio of liposome components, adequate size, molecular weight, and N/P ratio (the ratio of cationic amine groups to anionic phosphates of NA).^104^ These parameters were shown to influence both the transfection efficacy and cytotoxicity. While the molecular weight of a carrier can be increased through incorporation of ligands thus enhancing its specificity and serum resistance, large liposomes demonstrate low transfection rate and relatively high cytotoxicity.^105^, ^106^ High N/P ratio was reported to improve carrier‐cell interaction (due to high positive charge of the liposome), however, such lipids showed increased cytotoxicity.^107^, ^108^ To date, various types of lipid carriers have been described that are characterized by different lipid composition and parameters of transfection efficacy. The next step in improving the nanocarrier design will be the use of specific mitochondria‐targeting molecules.^89^, ^109^, ^110^

## 9 CURRENT MITOCHONDRIAL GENE DELIVERY APPROACHES

Notably, there are currently other potential strategies for gene therapy being developed, such as viral vectors, CRISPR/Cas9, and stem cells–based therapy. In spite of significant efficacy and convenience for some purposes, these methods are not flawless and have obstacles to overcome before application to mitochondria.^111^ Viral‐based methods, although demonstrate high transfection efficiency, are prone to initiate host immune response and have a limit in size of delivered genes.^112^ CRISPR/Cas9, which is a well‐known method of nuclear genome editing, still remains uncertain for the mitochondrial genome. There is complication of guide RNA import to these organelles as well as unwanted total mtDNA reduction under action of cleaving enzymes.^113^ Stem cells are frequently discussed in cardiovascular disease treatment and can be observed in many studies as an effective therapeutic approach. However, obviously more researches in this area are needed because of wide diversity of stem cell available with unknown signaling pathways and genetic instability.^114^ Moreover, a lot of complications are related to high costs and difficulties in maintaining cell culture.^115^ In comparison to abovementioned strategies, lipid‐based gene delivery has the main advantage of various design facilities, thus making it available to be customized and structured according to the purpose and also suitable for mitochondria targeting.

Early techniques of mitochondria‐specific lipid‐based NA carriers creation included the use of DQAsome (dequalimium chloride), early MITO‐porter, and STPP‐L (stearyl triphenylphosphonium‐modified liposomes). These approaches demonstrated relatively low specificity, substantial cytotoxicity, and, in some cases, low transfection efficacy, which considerably limited their use.^116^, ^117^, ^118^ Further development of nanocarriers employed modifying their biocompatibility and incorporating certain mitochondria‐specific molecules.^119^

In order to improve biocompatibility, new variations in liposomes were designed and tested in vitro and in vivo. TPP‐based carriers were incorporated with PGE‐PE (polyethylene glycol‐phosphatidyl ethanolamine) polymers which managed to decrease cytotoxicity and increase the lifetime of the carriers in blood serum.^120^ MITO‐Porter was modified with S2 peptide (Dmt‐D‐Arg‐FK‐Dmt‐D‐Arg‐FK‐NH2) and showed improved cell viability rate.^121^ At the same time, attempts were made to increase specificity of the carriers by supplementing them with mitochondria‐fusogenic lipids and targeting molecules.^103^

Currently, lipid‐based carriers for gene delivery can provide for a wide range of therapeutic effects for mtDNA damage correction in different states, including atherosclerosis. Moreover, these particles are characterized by specific and accurate action in comparison to currently approved treatment methods, thus avoiding adverse effects. In relation to atherosclerosis, such targeted therapy may correct the mtDNA damage, which currently cannot be repaired with any other available tools, and thus alleviate the mitochondrial dysfunction.

Although lipid‐based gene delivery to macrophage mitochondria represents a novel technology in atherosclerosis prevention and treatment, there is still a serious lack of studies further characterizing this approach in vivo. It is also worth mentioning that liposome‐based atherosclerosis therapy had been discussed before. However, no clinical trial results are available to be observed at the moment because of problems needed to be solved first. Most notably, the problem of inequality between cell cultures, animal model and human, hence there is always a difference in structure, morphology, and biochemistry of the plaque.^122^, ^123^ This fact, on the one hand, can alter expected cytotoxicity, and, on the other hand, explains the importance of searching for efficient plaque macrophage targets. Another problem is distribution and plaque targeting due to interactions with plasma proteins and high hemodynamics in the arteries. Unlike NP‐based cancer therapy, the carriers targeting atherosclerotic plaques cannot be administered locally due to plaques presence in many vessels. That is why DNA vector and the carrier must remain intact from serum components to achieve proper bioavailability, reach mitochondria, and interact with mtDNA. In this case, a number of solutions in carrier design have been suggested, and some of them are currently being tested in models. Moreover, there is always a question of cost effectiveness related to nanomaterials and carrier preparation.^124^ Today we can conclude that more preclinical studies are needed to reach the step of clinical approval of this promising therapeutic approach.

## 10 CONCLUSION

Converging evidence identifies macrophages as key players in atherosclerosis pathogenesis and, consequently, as potential therapeutic targets. Macrophage mitochondria, in particular, appear to be interesting from the point of view of future therapies development, as mtDNA damage is associated with the pathology development. Lipid‐based nanocarriers may provide a solution for targeted gene delivery into macrophage mitochondria to alleviate atherosclerosis‐associated mitochondrial dysfunction and oxidative stress. These agents are characterized by high transfection efficacy and low cytotoxicity. More studies are needed, however, to translate the results obtained in in vitro experiments to clinical practice.

## CONFLICTS OF INTEREST

The authors declare no conflict of interest.

## AUTHORS’ CONTRIBUTION

FHZ wrote the manuscript text; all other authors contributed to the manuscript development and review.
